# Constraining the preservation of organic compounds in Mars analog nontronites after exposure to acid and alkaline fluids

**DOI:** 10.1038/s41598-020-71657-9

**Published:** 2020-09-15

**Authors:** Carolina Gil-Lozano, Alberto G. Fairén, Victoria Muñoz-Iglesias, Maite Fernández-Sampedro, Olga Prieto-Ballesteros, Luis Gago-Duport, Elisabeth Losa-Adams, Daniel Carrizo, Janice L. Bishop, Teresa Fornaro, Eva Mateo-Martí

**Affiliations:** 1 Centro de Astrobiología, CSIC-INTA, Madrid, Spain; 2 Departamento de Geociencias Marinas, Universidad de Vigo, Vigo, Spain; 3 Department of Astronomy, Cornell University, Ithaca, NY USA; 4 SETI Institute, Mountain View, CA USA; 5 INAF-Astrophysical Observatory of Arcetri, Florence, Italy

**Keywords:** Astrobiology, Geochemistry, Mineralogy

## Abstract

The presence of organic matter in lacustrine mudstone sediments at Gale crater was revealed by the Mars Science Laboratory Curiosity rover, which also identified smectite clay minerals. Analogue experiments on phyllosilicates formed under low temperature aqueous conditons have illustrated that these are excellent reservoirs to host organic compounds against the harsh surface conditions of Mars. Here, we evaluate whether the capacity of smectites to preserve organic compounds can be influenced by a short exposure to different diagenetic fluids. We analyzed the stability of glycine embedded within nontronite samples previously exposed to either acidic or alkaline fluids (hereafter referred to as “treated nontronites”) under Mars-like surface conditions. Analyses performed using multiple techniques showed higher photodegradation of glycine in the acid-treated nontronite, triggered by decarboxylation and deamination processes. In constrast, our experiments showed that glycine molecules were preferably incorporated by ion exchange in the interlayer region of the alkali-treated nontronite, conferring them a better protection against the external conditions. Our results demonstrate that smectite previously exposed to fluids with different pH values influences how glycine is adsorbed into their interlayer regions, affecting their potential for preservation of organic compounds under contemporary Mars surface conditions.

## Introduction

The abundance of hydrous minerals across the martian surface highlight the existence of early aqueous environments that could have supported life^1–6^. However, Mars currently has a very thin and dry atmosphere and lacks a global magnetic field that shields the surface from ionizing radiation, including most of the UV-C range (190–280 nm). Under this inhospitable environment, biosignature preservation is only expected within mineral matrices able to confer protection from the harsh surface conditions on Mars^7,8^. In this context, clay minerals, with a long residence time in soils and sediments, are particularly interesting on Mars because, in addition to serving as geochemical markers of past aqueous conditions, they are excellent reservoirs for preserving organic compounds over geological time scales^9^. In particular, smectites present an expandable 2:1 sheet structure suitable for hosting small organic molecules (e.g., amino acids), and can act as a shield against extreme environmental conditions. These properties are well known on Earth, where smectites are the major reservoirs of carbon in marine sediments^10^, and previous investigations have shown their capacity to preserve organic molecules under simulated Martian conditions^11–14^.

Since 2012, the Curiosity rover has been characterizing the geological setting of the Crater Gale, which is composed of ancient fluvio-lacustrine sediments with potential past habitability^15–19^. Analysis performed with the Sample Analysis at Mars (SAM) instrument suite, on board the Curiosity rover, identified the presence of chloride- and sulphur-rich organic compounds on drills samples collected from (i) the Sheepbed member at Yellowknife Bay^20^, and (ii) Pahrump Hills at the base of the Murray formation^21^, respectively. The successful detection of organic compounds for the first time on Mars indicates that it is possible to identify them within favorable mineral reservoirs even under the inhospitable conditions of the surface of Mars. These sedimentary units contain both smectites and organic compounds, and present evidence of multiple episodes of aqueous alteration processes^19,22,23^. In particular, X-Ray Diffraction (XRD) data obtained from the CheMin instrument revealed the presence of trioctahedral smectite in the Cumberland sample (CB) from the Sheepbed member unit^24^. This smectite is thought to be formed by isochemical aqueous alteration of detrital olivine under neutral to moderate alkaline pH, and anoxic to poorly oxidizing conditions^22,25^. Its basal spacing of 13.2 Å suggests that this is a divalent saturated smectite with an interlayer cation of high hydration energy (e.g., Mg^2+^) or a partially chloritized smectite^25^. The detection of Ca-sulfates in voids and hollow nodules placed in the same sedimentary unit suggests the occurrence of two distinct diagenetic fluid events. In the case of the Mojave sample (MJ2), from the Pahrump Hills unit, the clay mineral identified shows a broad basal peak near 10 Å, consistent with a poorly crystalline collapsed smectite or an illite^19^. The identification of jarosite and hematite in this sedimentary unit indicates more acidic and oxidizing weathering conditions^22^. Mineralogical differences between both sedimentary units include the transition from magnetite to hematite and the increasing abundances of sulfates, reflecting a change in the environmental conditions (from more alkaline and reducing towards more acidic and oxidizing conditions)^22^.

The importance of clay minerals as targets for biosignature preservation has been widely recognized^7,11–14,26–30^ but, to the best of our knowledge, there is no previous works analyzing the particular effect of the type of activation, alkaline or acidic, on the efficiency of clays as preservers of organic compounds under Mars-like surface conditions. The exposure to different diagenetic fluids can induce changes in the smectite structure (e.g., surface area and layer charge) that might affect chemical interactions with organic molecules and compromise its preservation capacity on the long-term.

Here, we investigated whether the exposure to distinct external fluids (i.e., alkaline and acid) can affect the organic preservation capacity of nontronites under Mars-like conditions (PCO_2_ atm ~ 7 mbar and high fluxes of UV radiation) at the Planetary Atmospheres and Surfaces simulation Chamber (PASC)^31^ using glycine as a biomarker (see “Methods”). Previous studies confirmed that glycine is rapidly degraded under simulated Martian conditions^32,33^, but its destruction rate was substantially reduced when it was embedded in nontronite^13^. We characterized nontronite-treated samples spiked with glycine combining different techniques (i.e., Powder X-ray Diffraction [PXRD], Diffuse Reflectance Infrared Fourier Transform Spectroscopy [DRIFTS], X-ray Photoemission Spectroscopy [XPS], Raman and Scanning Electron Microscope [SEM]) in order to identify diagnostic features to better understand the most important organic-clay interactions before and after exposure to Mars-like conditions (see “Methods”). We also analyzed the in-situ Raman spectra of glycine-nontronite samples monitored during the exposure time. Data derived from these simulation experiments can help to constrain the target mineralogy to successfully detect biosignatures and highlight the need to go further in the comprehension of clay organic preservation under Mars-like conditions.

## Results

### Samples characterization

**Figure 1:**
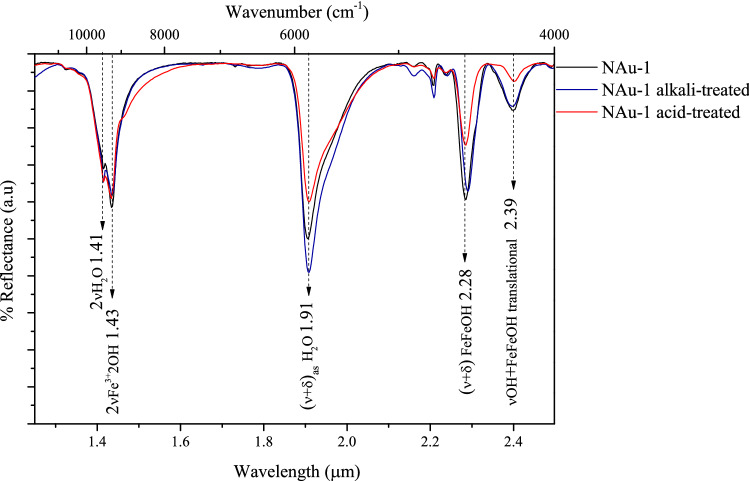
Near-infrared spectra of nontronite treated samples. Major bands and molecular vibration assignation are highlighted with arrows in the figure (ν, stretching; δ, bending; as, asymmetric).

Near-InfraRed (NIR) spectra of both treated clays show slight differences between them (Fig. 1). The spectrum of the acid-treated nontronite shows a decrease in intensity of the hydroxyl stretching, bending and translation modes associated with ferric iron^34^ (FeFeOH), reflecting a partial dissolution of octahedral cations^35,36^, which are not observable in the alkaline-treated nontronite. X-ray element distribution maps of both treated clays show differences also in the Si/O ratio, which is lower in the acid-treated than in the alkali-treated, 3.58 vs. 3.90, respectively (Supplementary Fig. S1). In addition, the amount of the Na interlayer cation diminishes in the acid-treated samples to a value near to zero. This could indicate H^+^ exchange for Na^+^ in the interlayer sites.

**Figure 2:**
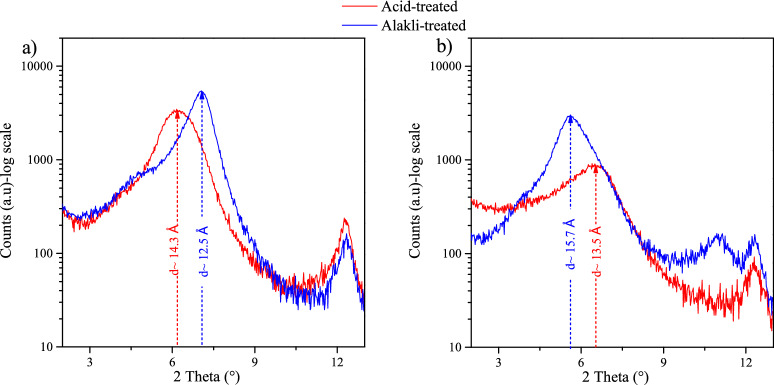
XRD patterns of oriented aggregates, air-dried, showing the basal reflections of treated nontronites, (**a**) without glycine and, (**b**) after glycine incorporation.

The XRD pattern of purified and treated nontronites only shows nontronite peaks with trace amounts of kaolinite (Supplementary Fig. S.2). The basal d(001) reflection of oriented aggregate mounts became broader and less intense in the acid-treated clay compared with the sharp basal reflection of the alkaline treated clay (Fig. 2a), pointing to a loss of crystallinity after reaction with HCl^35^ (see “Methods”). The interlayer distance in alkali-treated samples at 12.5 Å is close to the typical value of mono-hydrated Na-smectite^37^, whereas in acid-treated samples this is shifted to 14.3 Å. The former basal spacing is more characteristic of a bi-hydrated smectite, suggesting that some Mg^2+^ leaching from the octahedral layer could be adsorbed at the interlayer.

The suspensions of glycine with nontronite treated samples have an equilibrium pH of 4.7 and 8.7 for acid- and alkali-treated nontronite, respectively. At these pH values glycine is in the zwitterionic form (NH_3_^+^–CH_2_–COO^−^), whereas nontronite can be positively and negatively charged^38^ according to the pH at zero point of charge (pHzpc), which is in the range from pH 6.5^39^ to pH 7.0^40^. Therefore, glycine can be adsorbed by electrostatic forces or incorporated in the interlayer by ligand exchange. Treated clays after spiking with glycine also showed differences in their basal spacing, but in this case, the interlayer distance was lower in the acid-treated (13.5 Å) than in the alkali-treated sample (15.7 Å) (Fig. 2b). These changes in basal spacing will be discussed below. These samples will be hereafter referred to as “gly-acNon” for the acid-treated and “gly-alkNon” for the alkali-treated nontronite.

### Glycine degradation after exposure to Mars-like surface conditions

**Figure 3:**
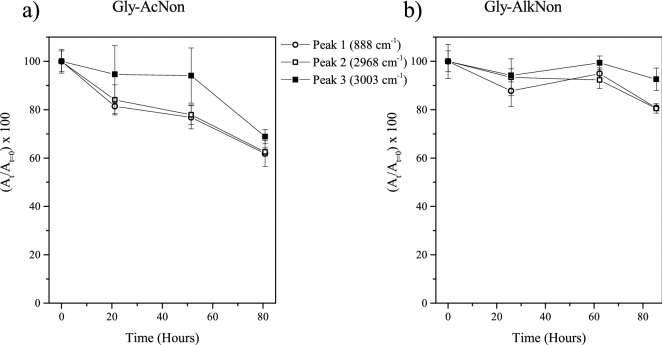
Integrated area variation of the peaks monitoring with Raman in situ analysis: (**a**) gly-acNon and, (**b**) gly-alkNon. We assigned these peaks to the stretching νCC + νCN at 888 cm^−1^ and, to the symmetric stretching, ν_s_CH, and asymmetric stretching, ν_as_CH, at 2,968 and 3,003 cm^−1^, respectively^42^. Bar errors represent the standard deviation of three consecutive spectra, variability in the spectra is due to technical limitations to improving the quality of the spectra.

Samples were monitored by Raman spectroscopy during the experiment to analyze in situ degradation of glycine under UV Martian surface conditions, for about 80 h. Due to the significant fluorescence and the weak Raman signal of clays^41^, we analyzed the most intense peaks of the organics (with the highest signal to noise ratio). The peaks corresponding to glycine functional groups appear at 888 cm^−1^ (stretching, νCC + νCN) and, at 2,968 (symmetric stretching, ν_s_CH) and 3,003 cm^−1^ (asymmetric stretching, ν_as_CH), respectively^42^. Upon exposition to Mars-like conditions, these peaks diminished simultaneously, more abruptly in the acid-treated nontronite (Fig. 3). Ex situ Raman spectra also revealed a higher degradation of the acid-treated clay. We focused on the same peaks of in situ Raman spectra at 888 cm^−1^ (νCC + νCN) and at 2,968 and 3,003 cm^−1^, (ν_s_CH and ν_as_ CH), and corroborated that the acid-treated clay showed a higher diminution of these peaks (Supplementary Fig. S.3).

**Figure 4:**
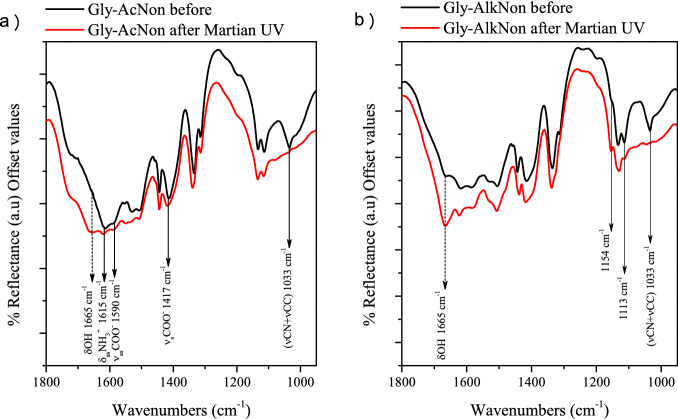
Infrared spectra in the 950–1,800 cm^−1^ region before and after exposure to UV Mars-like surface conditions, (**a**) in gly-acNon and, (**b**) in gly-alkNon samples. The solid arrows indicate an intensity decrease highlighting the most important changes of the glycine functional groups, dotted arrow shows an intensity increase of OH band.

Infrared spectra in the medium region were used to analyze major differences in the samples after exposure to UV radiation under Mars-like conditions. Spectra of glycine-clay pellets showed the bands assigned to NH_3_^+^ and COO^−^ groups of the zwitterion form of glycine molecules (^+^NH_3_CH_2_COO^−^) (Table 1). After UV exposition, the most remarkable changes reflect a decrease of the band at 1,033 cm^−1^, assigned to (νCN + νCC) in both clay samples (Fig. 4). However, gly-acNon also shows a pronounced decrease in the band at 1615 cm^−1^, assigned to an asymmetric bending of NH_3_ (δ_as_NH_3_), suggesting further organic degradation. Other minor changes include (i) a slight decrease in the intensity of COO^−^ at 1587 (asymmetric) and 1,415 cm^−1^ (symmetric) stretching bands in the gly-acNon sample; (ii) an increase of the bending mode of OH (at 1665 cm^−1^) in both treated nontronites; (iii) a change in the vibration bands at 1,113 and 1,154 cm^−1^ (intensity decrease at 1,113 cm^−1^ but increase at 1,154 cm^−1^) in the gly-alkNon sample.

**Table 1:** spectral assignements and wavenumbers of main glycine IR bands in the 1,000–1,800 cm^−1^ region.

Wavenumber (cm^−1^)		Band assignment
This work	Reference	
1033	1034	νCN, νCC
1113	1112	ρNH_3_
1130	1131	ρNH_3_
1311	1310	twCH_2_
1338	1334	ωCH_2_
1417	1413	νsCOO
1444	1444	δCH_2_
1507/1534	1505/1525	δasNH_3_
1590	1596	νasCOO
1625	1615	δasNH_3_

*ν* stretching, *ρ* rocking, *tw* twisting, *δ* bending, *ω* wagging.

Reference wavenumbers were taken from Rosado et al.^42^.

X-Ray Mapping (XRM) analysis of glycine-nontronite pellets also evidenced a slight diminution of the glycine signal (C + N contribution) in the acid-treated nontronite, whereas the organic content in the alkaline-treated clay remained practically constant after exposure to Mars-like conditions (Supplementary Fig. S.4). Density measurements of the pellets before and after simulation experiments also showed a higher decrease in the gly-acNon sample, which showed a density reduction of 26% versus the 18% registered in the gly-alkNon sample.

**Figure 5:**
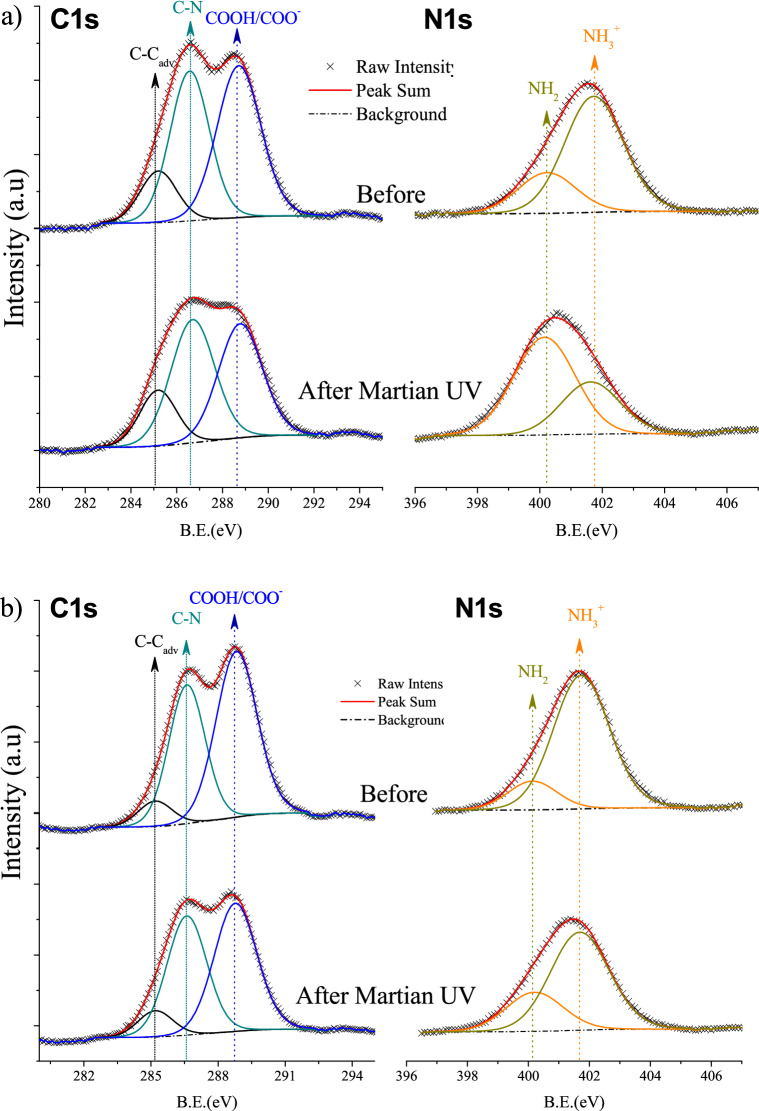
XPS analysis of C1s and N1s orbitals of glycine-nontronite pellet samples before and after exposure to Martian UV atmospheric conditions (**a**) gly-acNon and, (**b**) gly-alkNon, samples.

Detailed surface analysis performed with XPS provides a better understanding of the glycine-clay interaction and glycine degradation in our experimental settings. Orbitals assigned to clay structure (e.g., Si2p, Fe2p) did not show any change after exposure to UV radiation, and therefore, we focused on the C1s, O1s and N1s orbitals to identify changes in the functional groups of glycine (Fig. 5). Both pellets showed a decrease in the component assigned to COO^−^/COOH at 288.8 eV^43^. It is possible to distinguish between the protonated COOH (~ 533 eV) and carboxylate COO^−^ form (~ 531.5 eV) of the carboxylic acid in the O1s region^44^. However, multiple inorganic species (e.g., oxydes, hydroxides), including water (~ 533 eV), fall in the narrow peak of O1s, therefore, the deconvolution of this region is challenging. The O1s spectra of the pellets showed a decrease in intensity after simulation experiments (Supplementary Fig. S6). In the glyc-acNon pellet, we also identified a slight decrease of the component at 286.6 eV, assigned to the C–N^43^, less evident in gly-alkNon. Regarding the N1s orbital, we identified the major contribution at 401.6 eV, assigned to NH_3_^+^^43^ in both glycine-Non pellets before exposure to UV radiation. After the experimental procedure, this component decreases in favor of the increase of the 400.2 eV peak assigned to NH_2_^43^, but it is noteworthy that this decrease is more pronounced in the gly-acNon.

These results clearly indicate that glycine is more rapidly degraded under Mars-like conditions when it is embedded in acid-treated nontronite.

## Discussion

Getting insights about the mineralogy that may host biomarkers on the surface of Mars is certanly a primary task for the upcoming ExoMars and Mars2020 rover missions. Nontronite, detected in several locations on Mars, has been proved to be very stable under the Martian surface^45–48^ and its high ferric iron content (Fe^3+^ is a strong UV absorber) has been related to its high potential of organic preservation^11,49^. In addition, this smectite shows the most diverse and favorable adsorption behaviors when compared to other clay minerals, being able to adsorb nucleotides at very high equilibrium concentrations^50^. We studied the degradation of glycine as a target molecule to constrain the capacity of treated-nontronites to preserve organic matter under Mars-like conditions. Previous investigations have demonstrated that glycine is very rapidly degraded by UV radiation; indeed, the half-life of this amino acid under a Martian-like UV flux is estimated to be just of a few tens of sols (around 250 h)^32,33,51–53^. However, the survival time of glycine under Mars-like conditions substantially expands when it is embedded into nontronite minerals^13^. Our results presented here show for the first time that subtle changes in the structure of nontronite, triggered by exposure to different fluids (acid versus alkaline conditions), will modify the protection that these Fe-smectite minerals provide to the organic matter under Mars environmental conditions.

In our experiments, we used short exposure times to acidic or alkaline fluids (approximately 2 h), in order to induce the clay activation (i.e., layer charge, basal space) but retaining most of their sheet structure. Overall, the exposure of clay minerals to acidic fluids, commonly referred to “acid activation”, involves the dissolution of the octahedral layers (M-OH) and results in an enrichment of amorphous SiO_2_ coming from the tetrahedral sheet. This process increases the specific surface area (SSA)^36,54^ providing more sites of binding, which in turn is expected to increase the affinity for organic molecules. During the acid treatment of nontronite, protons enter the clay structure, partially dissolving the octahedral layers, which is reflected in a decrease in the intensity of FeFeOH vibrations (Fig. 1). The loss of crystallinity observed in the basal reflection peak (Fig. 2), together with the increment of the Si/O ratio and the leaching of the interlayer Na cation (Supplementary Fig. S1), suggest the coexistence of unaltered layers with a gel-like SiO_2_ phase from the tetrahedral sheet, pointing to a partial amorphization of the acid-treated clay. On the other hand, the exposure of clay minerals to alkaline fluids induces the dissolution of silica, which can also generate an increase of the specific surface area^55^. The alkali-treated nontronite shows basal reflections sharper than the acid-treated one, indicating a higher regularity of its mineral structure (Fig. 2).

In principle, it is expected that the concentration of biomarkers on Mars would be low (if any). Here we used a high organic-mineral proportion (10^–3^ mol glycine/g of nontronite) that mimics the optimum ratio for glycine preservation embedded in nontronite based on the experiments performed by Poch et al.^13^. This ratio allowed us to ensure that glycine molecules are incorporated in the interlayer^56^ and discriminate the preservation potential of both treated clays after exposing them to UV-light under Mars-like surface conditions (around 80 h) by using complementary spectroscopies techniques. However, the high amount of glycine used can complicate the organic-clay interactions, because of physical interactions between the glycine molecules can also occur.

The presence of COO^−^ and NH_3_^+^ infrared bands in the glycine-clay pellets (Table 1) shows that glycine is in the zwitterionic form, in agreement with pH measurements of the glycine-clay equilibrated solutions. After glycine incorporation, the basal peak is shifted in both treated nontronites. In the acid-treated nontronite, this shift is towards lower d-spacings (from 14.3 to 13.5 Å), whereas in the alkali-treated is to higher d-spacings (from 12.5 to 15.7 Å). Ramos and Huertas^56^, studied the adsorption of glycine on K-montmorillonite at different pH values and glycine concentrations. They realized that the adsorption of glycine increases at acidic pH values at the clay surface and, only when the edge surface is saturated, the interlayer adsorption of glycine takes place. A reduction of the basal spacing is associated to the exchange of the solvated interlayer cation by one monolayer of glycine molecules. However, the increase of the basal spacing observed in the alkaline treated nontronite suggests that the edge adsorption was less effective, favouring the incorporation of multilayers of glycine molecules into the interlayer space^57^. One possibility that might explain the reduction of cation exchange capacity observed in the acid treated sample could be the formation of interstratified layers (HI) by an incipient process of aluminization^58,59^. Nevertheless, we think that the influence of this process should be negligible in our samples, because of their low aluminum content (which remains nearly constant after chemical treatments, Supplementary Fig. S.1) and the short exposition time to the acid solution (2 h).

Infrared spectra of glycine-clay pellets display bands of both nontronite and glycine functional groups whereas in Raman spectra (in situ and ex situ analysis), the organic signals are the most remarkable features. After samples exposure to Mars-like conditions, major changes in infrared spectra include an intensity decrease of the νCC + νCN component at 1,033 cm^−1^ in both pellets, and a further decrease of the δasNH_3_^+^ component at 1615 cm^−1^ in the gly-acNon (Fig. 4). During the UV irradiation inside the PASC chamber, in situ Raman peaks at 888 cm^−1^ (νCC + νCN) and at 2,968 and 3,003 cm^−1^ (νsCH_2_ and νasCH_2_) show a gradual diminution in the gly-acNon pellet, whereas in the gly-alkNon sample remain practically constant (Fig. 3). This trend was corroborated with ex situ Raman spectra (Supplementary Fig. S.3). Overall, the intensity decrease of IR and Raman bands after exposure to UV under Mars surface conditions points to a higher photodecomposition of glycine molecules in the gly-acNon pellet. We did not observe any fingerprint in IR or Raman spectra that we can relate to the formation of new by-products, and therefore, we suggest CO_2_ gas was the dominant photoproduct^51^.

Detailed surface analyses performed with XPS provide us a better understanding of the glycine-clay interaction and glycine photodegradation after the exposure of the samples to Mars-like conditions. An important point is that the clay structure did not show any change upon exposure to UV radiation (Supplementary Fig. S.6). Therefore, density reductions of the pellets after simulation experiments (26% in gly-acNon and 18% in gly-alkNon) are most likely due to a decomposition of glycine molecules by UV radiation. Besides, all the changes observed in the C and N core-level spectra can be assigned to glycine alteration. Both pellets showed a decrease of the COO^−^/COOH component at 288.8 eV (a reduction of about 25% vs 20%, in gly-acNon and gly-alkNon samples, respectively) in agreement with previous works that have identified decarboxylation as one of the main photodegradation pathways of amino acids^11,51^ (Fig. 5). We also observed a slightly reduction in the intensity of the component at 286.6 (± eV), assigned to the C–N group, which again is more evident in the gly-acNon sample, suggesting that deamination was also occurring. Interestingly, we identified a decrease of the NH_3_^+^ component (at 401.6 eV) and an increase of the NH_2_ contribution (at 400.2 eV) in both samples, being more noteworthy in the gly-acNon pellet. Previous works have shown that Al-smectites and Mg-smectites favored the accumulation of N-rich organic residues after exposing ANR under hydrothermal conditions^28,29^. In our experiments, the fact that the fragmentation of the glycine molecule (mainly driven by decarboxylation) and the increment of the NH_2_/NH_3_ ratio are more noteworthy in the acid-treated sample, might also indicate that glycine photodegradation produces an enrichment of amino-rich moieties over the samples, as previously suggested by Tzvetkov et al.^44^. However, we cannot rule out other chemical mechanisms such as the conversion of the zwitterions into neutral molecules due to charge transfer reactions^60^.

Even though acid-activation of clays is a widely known process to increase the adsorption capacity of these minerals^36^, our simulation experiments indicate that glycine is better preserved when is embedded in the less altered alkali-treated nontronite. In order to compare the preservation role of both treated nontronites, we estimate the quantum yields of photodissociation following the procedure described in Poch et al.^13^ (see Supplementary information). Our results show that the quantum efficiency of photodecomposition is reduced by a factor 1.3 in the alkaline treated nontronite, offering a higher photoprotective effect. In principle, the major difference between both treated clays resides on the partial amorphization and the leaching of interlayer Na^+^ from the basal space of the acid-treated clay. Therefore, these results show that glycine molecules were better intercalated into the interlayer space by cation exchange mechanism in the alkaline-treated nontronite, conferring them a better protection against the external radiation conditions. In terrestrial sediments, interlayer sites of smectites have been suggested to play a more determinant role than the external surface area (conventional BET) in preserving organic compounds^61^ and in retaining organic pollutants in clay barrier systems^62^. Our results highlight the role of the interlayer space to stabilize organic molecules under present-day Mars surface conditions, showing that subtle chemical variations in the hosting smectite could be key for the preservation of biomarkers in the long-term. These results are consistent with the work of Vinogradoff et al.^30^ that showed that the starting compositional nature of phyllosilicates (Al vs Fe-rich) strongly determines the chemical evolution of organic matter in asteroids. On the other way around, recent investigations have also revealed the influence of post-depositional hydrothermal alteration in the preservation of organic compounds in smectites and on the final mineral assemblages^28,29^. All these works highlight the importance of studying in detail the nature of the host minerals and their chemical evolution as the key to improve the searching for traces of life beyond Earth.

In Yellowknife Bay, the SAM instrument onboard the Curiosity rover identified organic compounds in the Cumberland drill hole (CB), but not in John Klein (JK), despite the proximity of both sites (2.75 m)^20^. The main mineralogical difference between both drill holes is associated with the peak assigned to the (001) basal spacing of a presumable trioctahedral smectite, which varied from 13.2 Å in CB to 10 Å in JK. However, the basal space of CB clays does not fit well with a collapsed smectite likely to find under the low RH conditions inside the CheMin. Therefore, several hypotheses have emerged to explain this interlayer space^17,24,25^, including the presence of divalent saturated smectite (e.g., Mg^2+^, Ca^2+^) with higher hydration energy to retain H_2_O molecules. In the same way, this divalent saturated smectite could have also better stabilized chemical interactions with organic molecules, in agreement with the divalent cation-bridging theory (DCBT)^63,64^. Results from a recent study indicate that the Yellowknife Bay sediments would have interacted with different fluids in an early post-depositional stage^23^. In this sense, we have shown here that the interlayer space of nontronite, which is very sensitive to external fluids, is a key variable to understand the preservation of organic compounds in the long-term under current Mars surface conditions.

## Conclusions

To help identifying the molecular remains of extinct life on Mars, laboratory analogues are well suited to guide the robotic search of biomarkers by the forthcoming rovers, NASA’s Mars 2020 Perseverance and ESA’s 2022 Rosalind Franklin. In this work, we analyzed the preservation of glycine embedded in activated notronites (previously exposed to acid and alkaline fluids) under Mars-like surface conditions. Our results show that subtle changes in the interlayer space of nontronites can modify the capacity of these Fe-smectite minerals to preserve organic matter. Our simulation experiments evidenced certain photodegradation of glycine molecules embedded in nontronite-treated minerals, triggered by decarboxylation and deamination processes after exposure to 80 h of UV radiation under Mars-like surface conditions. These changes are far more evident in the acid-treated nontronite, in which the interlayer space is partially lost by the formation of a gel-like silica phase. Conversely, physico-chemical interactions of glycine molecules are stronger in the alkali- treated nontronite, suggesting that the inclusion of glycine in the nontronite interlayer space was more efficient after alkaline activation than in the acidic case. Our results show that a short-term exposure to diagenetic fluids can modify the long-term preservation of biomarkers embedded in clays on the surface of Mars.

## Methods

### Sample preparation

Nontronita, NAu-1, was purchased from the Clay Mineral Society. Its chemical composition is (%): SiO_2_: 53.33 Al_2_O_3_: 10.22, Fe_2_O_3_: 34.19, MgO: 0.27, CaO: 3.47, Na_2_O: 0.08, K_2_O: 0.03, with chemical formula M^+^_1.05_[Si_6.98_Al_1.02_] [Al_0.29_Fe_3.68_Mg_0.04_] O_20_ (OH)_4_^65^. Initial clay samples were ground in an agate mortar and dispersed in sodium hexametaphosphate solution (NaHMP 5 wt%), stirring during 24 h. Clay fraction < 2 µm was obtained by low-speed centrifugation according to Stokes’ law. The suspension was titrated with acetate buffer (pH 5.0) until obtaining a pH of 6.8 (the sample was stirred for one more hour during which the pH remained stable) to remove carbonates. Then, the solid clay fraction (obtained by centrifugation) was washed with HPLC-grade water to remove soluble salts by subsequent cycles of sonication and centrifugation and dried in an oven 70 °C (approximately 24 h).

Purified nontronite was exposed to acid (HCl, 2.5%V/V) and alkaline (NaOH 2 M) solutions, respectively, during 2 h and subsequently washed with HPLC water and dried as in the purification treatment.

Glycine (purity ~ 99%) was purchased from Sigma Aldrich. Treated nontronites were then exposed to a glycine solution (0.1 M) under magnetic stirring during 24 h and dried in an oven at 70 °C (approximately 48 h) afterwards, to simulate the desiccation of ponds in a drying Mars^66^. We obtained an organic-mineral ratio of 10^–3^ mol glycine per gram of nontronite, similar to the ratio that showed the highest preservation capacity in the experiments performed by Poch et al.^13^. Then, we prepared pellets of each mineral sample spiked with glycine (gly-acNon and gly-alkNon) by pressing at 10 Ton, with a thickness of approximately 1 mm, in order to introduce them in the simulation chamber. Organic compounds are expected to incorporate in clay minerals during sediment burial diagenesis, but simulating these conditions in the laboratory is challenging. Therefore, we followed this step-procedure that allowed us to identify differences in the treated nontronites before glycine incorporation and to start with the same mass ratio of glycine per gram of nontronite before exposing them to Mars-like surface conditions.

### Planetary atmospheres and surfaces simulation chamber (PASC) and UV irradiation

Laboratory simulations of Mars-like conditions were performed in the PASC at the Centro de Astrobiologia (CAB). Pellet samples were placed in a gold sample holder perpendicular to the UV lamp (Hamamatsu C3150). To simulate Mars conditions, the chamber was set to reach a vacuum level of about 10^–5^ mbar in order to remove the atmospheric gases. Then, the gas mixture to simulate the Mars atmosphere (mainly composed of 99% CO_2_ and 0.6% H_2_O) was used to raise the partial pressure of the chamber up to 7 mbar. The UV radiation from the deuterium lamp enters the system through a quartz window. The UV flux measured at the sample position, obtained by integration of the irradiance over the 200–400 nm wavelength range, is 2.3 × 10^14^ photons/cm^2^ s, which corresponds to F = 2.3 × 10^14^ (6 eV photons) cm^−2^ s^−1^, about 10 times lower than the UV flux on the Martian surface. Further details about the technical characteristics of the UV lamp and the chamber are described elsewhere^31^. Additionally, the simulation chamber is equipped with a Raman spectrometer allowing in situ characterization of the samples under study. This Raman in situ analysis were carried out with a BWN-532-100E DPSS green laser (532 nm), spectral width 0.1 nm (typical 0.05 nm), power 100 mW. The laser is connected with the simulation chamber by an optical fiber probe and the scattered signal is detected with a BTC675 Exemplar Pro spectrometer, thermoelectrically cooled with backscattered CCD array, configured with 25 µm entrance slit and 1800 l/mm diffraction granting and 495 nm internal filter.

### Analytical techniques

Nontronite samples were characterized after exposure to acid and alkaline fluids by PXRD, DRIFTS and SEM. Chemical changes of pellets of minerals spiked with glycine before and after exposition to UV radiation under Mars-like conditions (about 80 h) were characterized by PXRD, Raman, DRIFTS, XPS and SEM. Previous studies have shown that nontronite is particularly effective in the absorption of UV radiation (200–400 nm) due to its high ferric content^11,67^. Therefore the spectroscopy techniques used here, with a penetration depth limited to the most surficial layers, in the range of nm (with the XPS) to the range of microns (with Raman, IR and EDX spectroscopies), are well suited to analyze the chemical changes accounted for absorption of the UV light.

PXRD was performed using a Bruker D8 Advance diffractometer with Cu Kα radiation (λ = 1.542 Å). Oriented powder XRD patterns were collected between 2° and 35° (2 theta) using a step size of 0.02°. DRIFTS spectra were collected with a Nicolet FTIR spectrometer using a DTGS-KBr detector at 2 cm^-1^ resolution in the MIR region (from 4,000 to 400 cm^−1^), with an XT-KBr beamsplitter and, in the NIR region (from 12,000 to 4,000 cm^-1^), with a Quartz beamsplitter. SEM images were performed with a JEOL JSM-5600 LV microscope equipped with energy-dispersive X-ray spectroscopy (EDX) INCA detector (20 kV) that allowed characterization of the morphology and the chemical composition of the samples, respectively. Raman ex situ analysis were performed with a Nd: YAG solid state laser with a wavelength of 532 nm non polarized, slit width of 200 µm. After focusing onto a monochromator (Horiba JobinYvon HRi550, 550 mm optical length), with a diffraction grating of 1,200 grooves/mm, the scattered light was detected with a Charge Coupled Device (CCD), 1,024 × 256 pixels, cooled to 203 K for thermal-noise reduction. The spectrometer was connected to a B&W Tek microscope with 20 × objective (Microbeam S. A., Spain) by fiber optics. XPS analyses of the samples were carried out in an ultrahigh-vacuum (UHV) chamber equipped with a hemispherical electron analyzer (Phoibos 150 MCD), using an Al Kα X-ray source (1,486.7 eV) with an aperture of 7 mm × 20 mm. The base pressure in the UHV chamber was 10^–8^ mbar, and the experiments were carried out at room temperature. A 30 eV pass energy was applied for acquisition of the overview sample, whereas a 20 eV pass energy was applied for the analysis of the following core level spectra: O 1 s, C 1 s, N 1 s, Na 1 s, Fe 2p, Si 2p and Al 2p. XPS spectra were analyzed using the CasaXPS software (version 2.3.21)^68^. Finally, an AccuPyc II 1340 pycnometer was used to analyze density variations in organic-clay pellet samples before and after simulation experiments.

## Supplementary information

Supplementary Information.
