# Supplementary material for: Constraining the preservation of organic compounds in Mars analog nontronites after exposure to acid and alkaline fluids
Source: Sci Rep. 2020 Sep 15;10:15097. doi: 10.1038/s41598-020-71657-9 (PMC7492362; doi:10.1038/s41598-020-71657-9)
Supplement: Supplementary file 1 — Supplementary Information. [file 41598_2020_71657_MOESM1_ESM.docx]

Supporting Information

# **Constraining the preservation of organic compounds in Mars analog nontronites after exposure to acid and alkaline fluids**

Carolina Gil-Lozano (1, 2)*, Alberto G. Fairén (1, 3)*, Victoria Muñoz-Iglesias (1), Maite Fernández Sampedro (1), Olga Prieto-Ballesteros (1), Luis Gago-Duport (2), Elisabeth Losa-Adams (2), Daniel Carrizo (1), Janice L. Bishop (4), Teresa Fornaro (5) and Eva Mateo-Martí (1)

(1) Centro de Astrobiología, CSIC-INTA, Madrid, Spain.

(2) Departamento de Geociencias Marinas, Universidad de Vigo, Vigo, Spain.

(3) Dept. of Astronomy, Cornell University, Ithaca, NY, USA.

(4) SETI Institute, Mountain View, CA, USA.

(5) INAF-Astrophysical Observatory of Arcetri, Florence, Italy

***** Corresponding authors: cgil@cab.inta-csic.es / agfairen@cab.inta-csic.es

The contents of this file include 6 figures (from S.1 to S.6), 1 table and 1 Appendix.

| **** |
| --- |
| Figure S.1: Chemical results of both treated nontronites obtained with the X-Ray mapping in SEM, a) atomic percentage composition of samples showing and a depletion of Na in the acid-treated nontronite. The O/Si ratio is lower in the acid-treated than in the alkali-treated (3.58 vs. 3.90, respectively) (C, from carbon taps, and Au, for gold coating, were not shown); b) phase maps composing by the signal contributions of Si (green), O (red) and Al (blue). |

|  |
| --- |
| Figure S.2: XRD patterns of randomly oriented powders of nontronite samples. Dashed arrows highlight the position of kaolinite reflections. The intensity of XRD was normalized to the basal peak to facilitate comparison. |

|  |
| --- |
| Figure S.3: Ex situ Raman spectra of two different spots of each pellet before and after exposure to UV martian surface conditions, (the surface of pellets were labelled in order to minimize spatial errors between measurements). Spectra show the peaks monitored in the in situ Raman analysis: a) gly-acNon and, b) gly-alkNon. |

|  |
| --- |
| Figure S.4: Atomic percentage composition of both glycine-nontronite pellet samples obtained with the X-Ray mapping in SEM, a) gly-acNon and b) gly-alkNon (since C K and N K peaks are very close and overlap in EDX spectrum, their contributions are put together; it is expected that a small contribution of the carbon signal comes from carbon taps). |

|  |
| --- |
|  |
| Figure S.5: Survey spectra of both glycine-nontronite pellet samples a) gly-acNon and b) gly-alkNon |

|  |
| --- |
| Figure S.6: Core level spectra of the gly-acNon (on the left) and on the gly-alkNon (on the right); a) Si 2p; b) Fe 2p and, c) O 1s. |

| Table S.1: fitting parameters for the core level C 1s and N 1s XPS spectra for Gly-acNon and Gly-alkNon samples. We used a Shirley for the background and a modified Lorentzian function for the line-shape. |
| --- |
| \| **C 1s** \| \| \| \| \| \| --- \| --- \| --- \| --- \| --- \| \| **Gly-acNon before** \| **B. E (eV)** \| **FWHM** \| **Area** \| **% Area** \| \| **C adv.** \| 285.2 \| 1.9 \| 1432.3 \| 13.2 \| \| **C-N** \| 286.6 \| 2.02 \| 4415.0 \| 40.7 \| \| **COO^-^/COOH** \| 288.7 \| 2.24 \| 5001.3 \| 46.1 \| \|  \|  \|  \|  \|  \| \| **Gly-acNon after UV** \| **B. E (eV)** \| **FWHM** \| **Area** \| **% Area** \| \| **C adv.** \| 285.2 \| 1.9 \| 1551.5 \| 16.6 \| \| **C-N** \| 286.7 \| 2.25 \| 4047.7 \| 43.2 \| \| **COO^-^/COOH** \| 288.8 \| 2.25 \| 3767.6 \| 40.2 \| \|  \|  \|  \|  \|  \| \| **Gly-alkNon before** \| **B. E (eV)** \| **FWHM** \| **Area** \| **% Area** \| \| **C adv.** \| 285.2 \| 1.8 \| 699.1 \| 6.8 \| \| **C-N** \| 286.6 \| 1.9 \| 4039.0 \| 39.5 \| \| **COO^-^/COOH** \| 288.8 \| 2.15 \| 5493.4 \| 53.7 \| \|  \|  \|  \|  \|  \| \| **Gly-alkNon after UV** \| **B. E (eV)** \| **FWHM** \| **Area** \| **% Area** \| \| **C adv.** \| 285.2 \| 1.85 \| 743.9 \| 8.3 \| \| **C-N** \| 286.6 \| 2.1 \| 3829.8 \| 42.7 \| \| **COO^-^/COOH** \| 288.8 \| 2.23 \| 4402.4 \| 49.0 \|  \| **N 1s** \| \| \| \| \| \| --- \| --- \| --- \| --- \| --- \| \| **Gly-acNon before** \| **B. E (eV)** \| **FWHM** \| **Area** \| **% Area** \| \| **NH_2_** \| 400.2 \| 2.19 \| 1854.2 \| 25.6 \| \| **NH_3_^+^** \| 401.7 \| 2.2 \| 5382.4 \| 74.4 \| \|  \|  \|  \|  \|  \| \| **Gly-acNon after UV** \| **B. E (eV)** \| **FWHM** \| **Area** \| **% Area** \| \| **NH_2_** \| 400.2 \| 2.3 \| 4696.2 \| 65.9 \| \| **NH_3_^+^** \| 401.6 \| 2.2 \| 2428.4 \| 34.1 \| \|  \|  \|  \|  \|  \| \| **Gly-alkNon before** \| **B. E (eV)** \| **FWHM** \| **Area** \| **% Area** \| \| **NH_2_** \| 400.2 \| 1.9 \| 1145.5 \| 15.8 \| \| **NH_3_^+^** \| 401.7 \| 2.15 \| 6100.4 \| 84.2 \| \|  \|  \|  \|  \|  \| \| **Gly-alkNon after UV** \| **B. E (eV)** \| **FWHM** \| **Area** \| **% Area** \| \| **NH_2_** \| 400.2 \| 2.21 \| 1810.4 \| 27.9 \| \| **NH_3_^+^** \| 401.7 \| 2.25 \| 4678.6 \| 72.1 \| |

**Appendix 1: Quantum efficiency of photodecomposition (following the procedure by Poch et al., 2015)**

In a photodegradation process, the quantum efficiency of photodecomposition Φ of a molecule is the probability of dissociation of this molecule after the absorption of a photon. This can be related to the ratio between photodissociated molecules ($N_{photodissociated molecules}$) divided by the number of photons absorbed by the molecules ($N_{absorbed photons}$) during the irradiation:

$$\Phi= \frac{N_{photodissociated molecules}}{N_{absorbed photons}}$$

$N_{photodissociated molecules}$ is calculated as follows:

$$N_{photodissociated molecules}=N_{0}-N_{final}=N_{0}\times(1-\frac{N_{final}}{N_{0}})$$

where $N_{0}$ and $N_{final}$ are the initial and final number of molecules constituting the deposit, respectively, before and after the irradiation. The ratio $\frac{N_{final}}{N_{0}}$ is determined by the integrated area of the carboxyl group determined by X-ray photoelectron spectroscopy (XPS), and $N_{0}$ is calculated assuming a rectangular shape of the organic-mineral deposit analyzed with XPS:

$$N_{0}=\frac{h \cdot l \cdot e\cdot d\cdot N_{A}}{M}$$

where $h \cdot l$ is the area exposed to the UV flux (m), $e$ is the thickness of the deposit analyzed by XPS, which can be approximate to the penetration depth of this technique (m), $d$ is the molecular density (g cm^-3^), $M$ is the molar mass of the molecule (g mol^-1^), and $N_{A}$ is the Avogadro’s constant (mol^-1^). The molecular density for glycine is 1.61 g cm^-3^ (Houck, 1930).

$N_{absorbed photons}$ is given by:

$$N_{absorbed photons}=\int_{t=0}^{t_{final}} \int_{\lambda=200 nm}^{\lambda=250 nm} \left( 1-e^{-k_{\lambda}e(t)} \right)\cdot F_{0}\cdot\pi\cdot R^{2}\cdot dt\cdot d\lambda$$

Where $F_{0}$ is the incident photon flux on the top of the deposit (photon cm^-2^ s^-1^ nm^-1^), $k_{\lambda}$ is the absorption constant of the molecule (m^-1^), and $e\left( t \right)$ is the thickness of the deposit at $t$ (m). Because the latter parameters cannot be satisfactorily accounted for, we have calculated the number of incident photons rather than the absorbed photons for our calculation of $\Phi_{exp}$:

$$N_{incident photons}=\int_{t=0}^{t_{fin}} \int_{\lambda=200 nm}^{\lambda=400 nm} F_{0}\cdot h \cdot l \cdot dt\cdot d\lambda$$

$$N_{incident photons}=F_{0 (200-400 nm)}\cdot h \cdot l \cdot\Delta t$$

$$\Phi_{exp}= \frac{N_{photodissociated molecules}}{N_{incident photons}}$$

With our experimental values, the quantum efficiencies of photodecomposition $\Phi_{exp}$ are of the same order of magnitude than the values reported by Poch et al. (2015), using the same mol per gram of mineral (see table below).

| \| **Sample** \| **Quantum efficiency of photodecomposition (molecule/photon)** \| **References** \| \| --- \| --- \| --- \| \| Glycine \| 6.3 x 10^-3^ * \| Poch et al. 2014 \| \| Glycine + Nontronite \| 2.4 x 10^-4^ * \| Poch et al. 2015 \| \| Glycine + acid-treated nontronite \| 4.7 x 10^-5^ ** \| This work \| \| Glcyine + alkali-treated nontronite \| 3.6 x 10^-5^ ** \| This work \| |
| --- | --- | --- | --- | --- | --- | --- | --- | --- | --- | --- | --- | --- | --- | --- | --- |
| * Integrated flux value between 200-250 nm, temperature 218 K  ** Integrated flux value between 200-400 nm, temperature 295 K |

**References**

Houck, R.C. (1930). A Note on the Density of Glycine. Journal of the American Chemical Society 52, 2420-2420.

Poch, O., Kaci, S., Stalport, F., Szopa, C. and Coll, P. (2014) Laboratory insights into the chemical and kinetic evolution of several organic molecules under simulated Mars surface UV radiation conditions. Icarus 242, 50-63.

Poch, O., Jaber, M., Stalport, F., Nowak, S., Georgelin, T., Lambert, J.-F., Szopa, C. and Coll, P. (2015) Effect of Nontronite Smectite Clay on the Chemical Evolution of Several Organic Molecules under Simulated Martian Surface Ultraviolet Radiation Conditions. Astrobiology 15, 221-237.
